# Hypertensive Retinopathy Secondary to Anlotinib Treatment

**DOI:** 10.3389/fphar.2020.00843

**Published:** 2020-06-05

**Authors:** Xiaohua Zhang, Li Peng, Qing Xie, Qingjing Wu, Xia Sheng

**Affiliations:** Department of Ophthalmology, Central South University Xiangya School of Medicine Affiliated Haikou Hospital, Haikou, China

**Keywords:** anlotinib, hypertensive retinopathy, high blood pressure, drug, treatment

## Abstract

**Purpose:**

We report a case of a middle-aged woman who developed hypertensive retinopathy following oral administration of Anlotinib.

**Observations:**

A 48-year-old woman presented to our hospital with sudden painless loss of vision in both eyes combined with headache, nausea, and vomiting following oral administration of Anlotinib. This drug is often used to control cancer progression. Due to the deterioration of her blood pressure, which reached 167/113 mm Hg, Anlotinib was discontinued and the blood pressure was controlled by hypertension medications. This normalized her blood pressure, alleviated headache, and restored her vision. She visited our eye department 37 days later for eye check-up. The best-corrected visual acuities was 0.3 in the right eye and 0.4 in the left eye. The fundus examinations revealed a clear boundary of the optic papilla with significant stellate exudation in the macular area. The posterior pole of the retina displayed high hemorrhage, with a cotton-wool spot appearance. Optical coherence tomography (OCT) revealed atrophy in the outer segment of macular area, and hard exudations in retinal layers. Based on these findings, hypertensive retinopathy was diagnosed, as a secondary complication of Anlotinib.

**Conclusions and Significance:**

Anlotinib can induce hypertensive retinopathy. Patients receiving this drug should be closely monitored for potential complications.

## Introduction

Majority of hypertensive retinopathy cases are caused by essential hypertension, also referred to as hypertension without an identifiable cause. Secondary hypertensive retinopathy is relatively rare in clinical practice, and is often caused by renal failure, toxemia of pregnancy, pheochromocytoma, and malignant hypertension (Kabedi et al., 2014). Drug-induced hypertensive retinopathy is far less common, and hypertensive retinopathy secondary to Anlotinib has not been reported.

Anlotinib is a novel oral receptor tyrosine kinase inhibitor (TKI) that mainly targets vascular endothelial growth factor receptor (VEGFR), fibroblast growth factor receptor (FGFR), platelet-derived growth factor receptors (PDGFR), and c-kit. In non-small cell lung cancer (NSCLC), metastatic renal cell carcinoma, and sarcoma, TKI produces favorable disease outcomes with only mild side effects (Shen et al., 2018). In May 2018, the China Food and Drug Administration (CFDA) approved Anlotinib as a third-line treatment for patients with advanced NSCLC.

Herein, we report a case of hypertensive retinopathy occurring secondary to oral administration of Anlotinib, accompanied with severe visual impairment.

## Case Description

**Figure 1:**
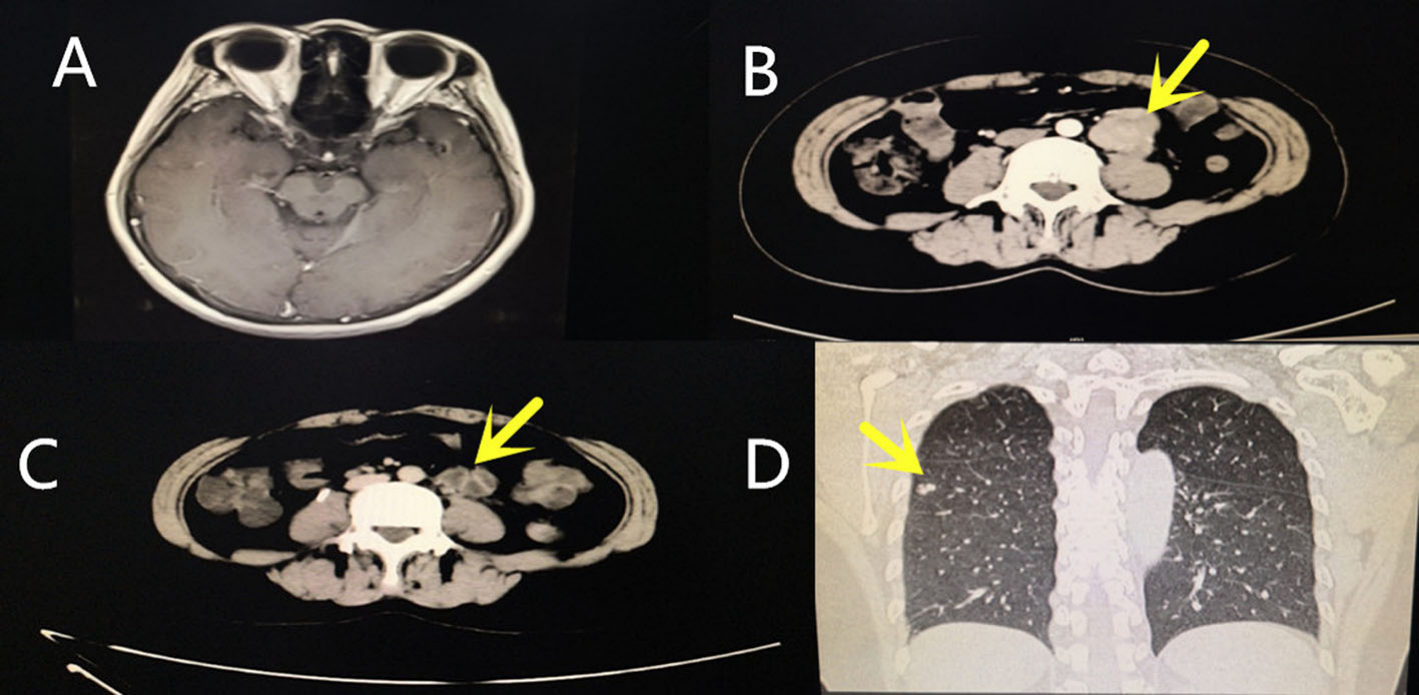
Multimodal imaging. **(A)** Brain MRI plain scan and enhancement. The brain MRI scan showed the brain was scattered with ischemic foci. **(B)** Abdominal three-dimensional CT and enhancements. The yellow arrow indicates the location of the primary retroperitoneal tumor. **(C)** Abdominal three-dimensional CT and enhancements. The yellow arrow indicates the location of the recurrent retroperitoneal tumor. **(D)** Chest CT. The yellow arrows indicate metastatic tumors in the lungs.

A written informed consent was obtained from the patient and her husband, who agreed to the publication of the patient’s identifiable data, such as age, race, gender, imaging data and hematological examination, and medical history. The study protocol was approved by the Institutional Review Board of Haikou People’s Hospital, and performed in accordance with the Declaration of Helsinki. The patient’s disease progression is summarized in **Table 1**. A 48-year-old woman without a history of ophthalmic complaints presented with a 1-month history of bilateral painless vision loss. The patient had no history of kidney disease and high blood pressure. She reported she had a sudden loss of vision in both eyes after the usage of Anlotinib (12mg once-daily, administered as 2 weeks on/1 week off, for about three months), when her blood pressure had reached 167/113 mmHg. At the time, she was on Anlotinib medication due to her metastatic leiomyosarcoma. She did not seek any medical help, but she stopped taking the medicine voluntarily because her oncologist had informed her of the potential high blood pressure as a side effect of the drug. Her blood pressure was measured twice a week, and her routine blood test was within normal limits: 90–110/57–80mm Hg. Three days later, her blurred vision worsened and she was unable to see near objects accompanied with severe headache, nausea, high blood pressure, and vomiting. Despite these symptoms, her consciousness was clear, her limbs were moving normally. She therefore visited our department of neurology for further treatment. The blood pressure was 164/109mm Hg, brain MRI plain scan and enhancement showed the brain was scattered with ischemic foci, without other special problems. (**Figure 1A**). She was, therefore, prescribed hypotension drug (angiotensin receptor blocker (ARB) Valsartan 160 mg once daily), circulation-promoting drug (Shuxuetong injection), and nerve-nourishing drug (Monosialotetrahexosylganglioside sodium injection). About a week later, her vision had improved with no symptoms of headache, vomiting, and her blood pressure returned to normal level (97/72mm Hg), even reached hypotensive levels (85/58mm Hg). Thus, Valsartan was stopped. Her blood pressure remained normal in the following days. One month later, she visited our eye department due to poor vision.

**Table 1:** The timeline of change in the patient’s condition.

Time	Event	Intervention	Outcome
On September 9 2016	Retroperitoneal leiomyosarcoma was found because of repeated abdominal pain (**Figure 1B**).	Simple surgical resection.	Abdominal pain was relieved after the tumor was removed.
On November 1 2018	Abdominal pain occurred again, and tumor recurred with pulmonary metastasis was found (**Figures 1C, D**).	Received combined chemotherapy regimen of MAID (Mesna, Adriamycin, Ifosfamide and Dacarbazine) for nearly 6 months. After this chemotherapy, she was put on oral Anlotinib, 12mg once-daily, administered as 2 weeks on/1 week off. Metoclopramide and omeprazole were taken orally to protect the stomach since chemotherapy.	Abdominal pain remission, the tumor was under stable control with no deterioration. Gastrointestinal discomfort occasionally occurred.
On September 24, 2019	Suffered a progressive sudden loss of vision in both eyes and blood pressure was markedly elevated. Three days later, her blurred vision worsened and had severe headache, nausea and vomiting.	She stopped taking Anlotinib voluntarily due to high blood pressure. Brain MRI showed the brain was scattered with ischemic foci. Thus, she was prescribed hypotension, circulation-promoting and nerve-nourishing drug from the department of neurology.	Blood pressure was well controlled. Vision improved from seeing near objects.
On October 31, 2019	Visited our eye department for unsatisfactory vision.	Ophthalmologic examinations were performed. Corrected visual acuity in the right eye was 0.3, and 0.4 in the left eye. Hypertensive retinopathy was diagnosed. Fufangxueshuantong and mecobalamin capsules were given to improve circulation and nourish nerves.	Gradual improvement in vision.
On November 9, 2019	Visited our eye department for review.	Ophthalmologic examinations were performed, corrected visual acuity in the right eye was 0.4, and 0.5 in the left eye, fundus condition was better than before. Continue to improve circulation and nutritional nerve treatment.	Vision slightly improved but couldn’t restore premorbid vision. The atrophy of the outer retina did not recover.

**Figure 2:**
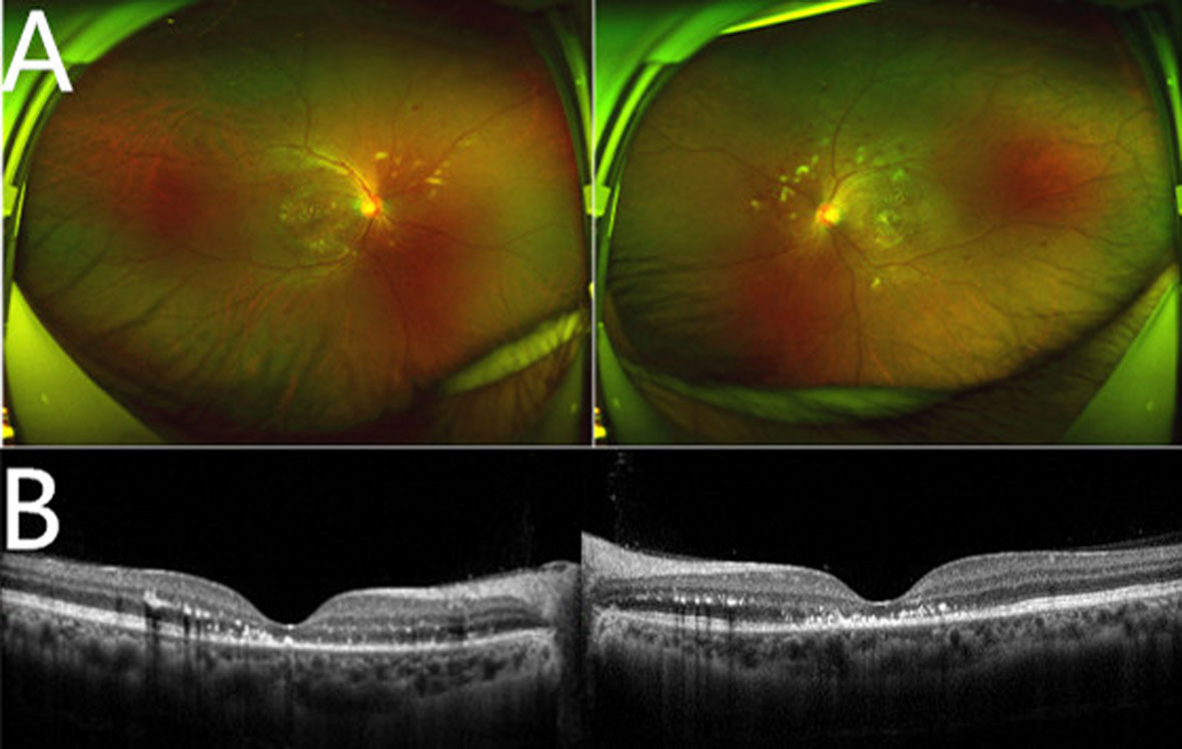
Multimodal imaging. **(A)** Ultra-wide field retinophotography of both eyes showing cotton-wool spots, widespread hemorrhage, and stellate exudation. **(B)** Horizontal Spectral Domain optical coherence tomography (SD-OCT) B-scan through the fovea of both eyes showing a regression of the exudative retinal detachment, ectoretina atrophy and hard exudations between the retinal layers.

**Figure 3:**
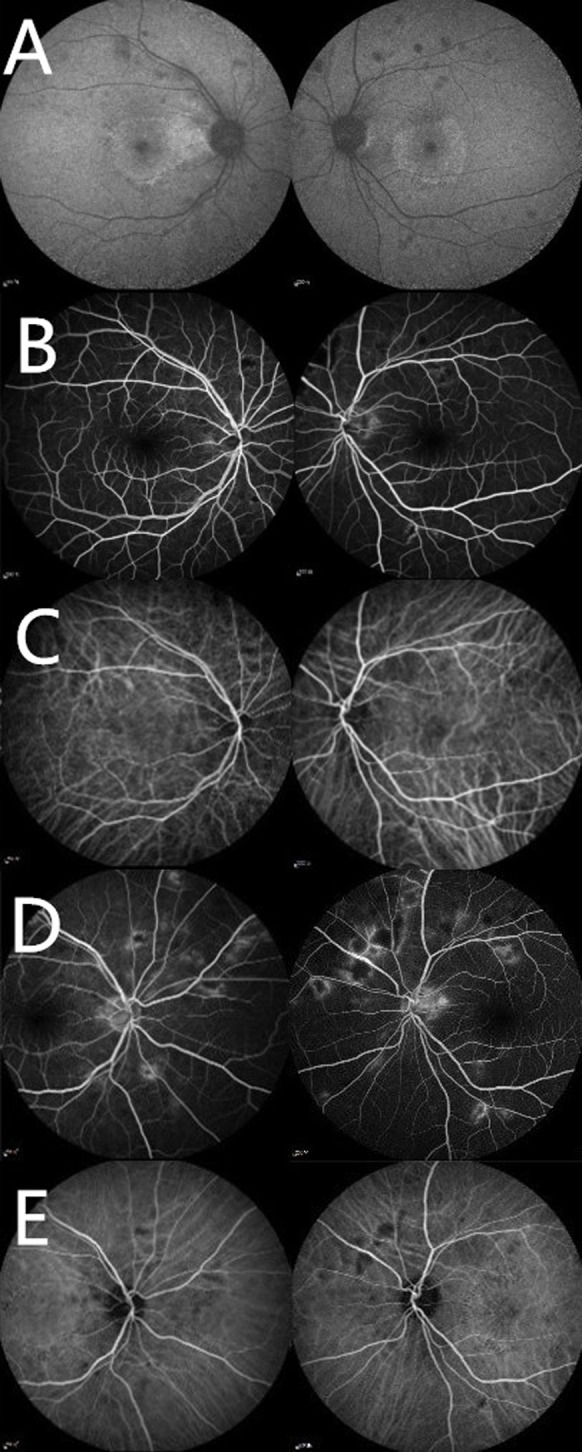
Multimodal imaging. **(A)** Fundus autofluorescence of both eyes revealing areas of hypo-autofluorescence due to retinal hemorrhages and cotton-wool spots, and annular hyper-autofluorescence in macular area due to hard exudation. **(B)** Early phase of Fluorescein Angiography (FFA) revealing areas of hypofluorescence. **(C)** Early phase of Indocyanine Green Angiography (ICGA) seeming normal. **(D)** Late phase of FFA showing hyperfluorescent spots. **(E)** Late phase of ICGA showing hypofluorescence areas.

Corrected visual acuity in the right eye was 0.3, and 0.4 in the left eye. Her ocular motility exam was normal. Intraocular pressure and the anterior section slit lamp examination showed no abnormal findings in both eyes. The fundus examination revealed the vitreous opacity, arterial narrowing, stellate exudation in macular area, widespread hemorrhage, and cotton-wool spots in retina. Horizontal Spectral Domain optical coherence tomography (SD-OCT) B-scan through the fovea showed ectoretina atrophy and hard exudations between the retinal layers, indicating possible exudative retinal detachment after occurrence (**Figure 2**). Fundus autofluorescence of both eyes revealed annular hard exudation in macular and areas of hypofluorescence due to retinal hemorrhages, cotton-wool spots, and retinal pigment epithelium (RPE) alterations. Fundus Fluorescein Angiography (FA) showed areas of hypofluorescence in the early phase which became hyperfluorescent in the late phase. Indocyanine green angiographyn (ICGA) was normal in the early phase and showed hypofluorescence areas in the late phase (**Figure 3**). Based on the patient’s history of elevated blood pressure, clinical manifestations, and relevant eye examinations, hypertensive retinopathy was diagnosed. Given that these symptoms occurred within month, the patient was now in the recovery stage. We therefore put her on fufangxueshuantong and mecobalamin capsules to improve circulation and provide nerves nourishment. About one week later, the patient came back, this time reporting a slight improvement of her vision, and the black shadow in front fluttered was less than before. The corrected visual acuity in the right eye was 0.4, and 0.5 in the left eye, but she still felt that her vision was not fully normal. The fundus examination revealed that the stellate exudation, hemorrhage and cotton-wool spots in retina were partly absorbed, compared to the last examination. SD-OCT scan through the fovea revealed that the atrophy of the outer retina did not recover. We asked her to continue her current treatments.

Her past medical history included an operation for retroperitoneal leiomyosarcoma in 2016. (**Figure 1B**). In November 2018, the tumor recurred with pulmonary metastasis, for which she received combined chemotherapy regimen of MAID (Mesna, Adriamycin, Ifosfamide and Dacarbazine) for nearly 6 months at Sun Yat-sen University Cancer Center. Apart from gastrointestinal discomfort, like nausea and vomiting, no other serious complications were present (**Figures 1C, D**). We were put on oral Metoclopramide and Omeprazole to protect the stomach since she was undergoing chemotherapy. After this chemotherapy, she was put on oral Anlotinib, 12 mg once-daily, administered as 2 weeks on/1 week off. The course of treatment was nearly three months. While taking the medicine, she went to Sun Yat-sen University Cancer Center for regular check-ups, such as blood, urine routine, blood biochemistry, thyroid function, coagulation, ECG, chest radiograph, abdominal B ultrasound, among other tests. The tumor was under stable control with no deterioration. Other than nausea and gastrointestinal discomfort, no other special abnormalities were present. Her blood pressure remained normal during this period. This medicine was stopped when she developed a sudden loss of vision in both eyes and high blood pressure.

## Discussion

We report an interesting and rare case of hypertensive retinopathy secondary to oral Anlotinib. This is the first report on ocular dysfunction caused by this drug. Poorly controlled high blood pressure affects the eye causing: retinopathy, choroidopathy and optic neuropathy. Hypertensive retinopathy occurs when the retinal vessels are damaged by elevated blood pressure. In such a case, retinal signs such as retinal flame-shaped and dot blot hemorrhage, hard exudate formation, necrosis of smooth muscle cells, and retinal ischemia occur forming of cotton-wool spots (Harjasouliha et al., 2017). Anlotinib 1-[[4-(4-fluoro-2-methyl-1H-indol-5-yloxy)-6-methoxyquinolin-7-yl]oxy] methyl] cyclopropanamine dihydrochloride) was manufactured by Chia-tai Tianqing Pharmaceutical Co., Ltd. in China. It is a novel multi-target TKI that is designed to primarily inhibit VEGFR2/3, FGFR1-4, PDGFR α/β, c-Kit, and Ret. In so-doing, it effectively suppresses tumor angiogenesis and growth, with acceptable toxicity as seen in lung squamous cell carcinoma, advanced lung adenocarcinoma, small cell lung cancer, soft tissue sarcoma, medullary thyroid cancer, and metastatic renal clear cell cancer (Han et al., 2018; Sun et al., 2018; Shao et al., 2019; Yang et al., 2019; Zhou et al., 2019). Its major side effects include fatigue, high blood pressure, hand-foot skin reaction, gastrointestinal reactions, triglyceride elevation cholesterol elevation, and blood thyrotrophin elevation (Sun et al., 2016; Shao et al., 2019; Wang et al., 2020). To rule out other factors contributing this patient’s hypertensive retinopathy, such as possible drug interactions, diet, and congenital factors, a detailed medical history was performed. Usually, she had a light diet and stable emotions, nothing special happened during that time. No other medicines were taken except for the stomach medicines and Anlotinib. None has reported that Metoclopramide and Prilosec can cause high blood pressure. After oral administration of Anlotinib, the patient experienced a sudden loss of vision accompanied with elevation of blood pressure, both of which were considered adverse reactions to the drug, leading to hypertensive retinopathy. The patient stopped taking the medicine voluntarily because her oncologist had informed her of the potential high blood pressure as a side effect of the drug, which would slowly return to normal once the drug is stopped. Her vision improved and blood pressure gradually returned to normal level after discontinuation of the drug and receiving blood pressure lowering treatment. One month after the onset of the disease, she returned to our ophthalmology department due to poor vision. Ophthalmologic examinations confirmed hypertensive retinopathy, which was in the recovery period. In fact, we speculated that the sudden loss of vision, headache, and increased blood pressure were the acute episodes of hypertensive retinopathy, but because she did not visit the eye clinic, hypertensive retinopathy was not diagnosed one month ago. Anlotinib administration has been reported to cause high blood pressure. High blood pressure is also a common adverse effect of VEGF pathway inhibitors (Pandey et al., 2018). In general, nearly all adverse reactions can be prevented and controlled by adjusting the dose, symptomatic supportive treatment, or suspending the medication. Anlotinib is a new anti-tumor drug which is expected to be widely used clinical practice. Thus, adverse reactions associated with it should be closely monitored. For patients with pre-existing high blood pressure, effective blood pressure control is advised before administering Anlotinib, and it should be routinely monitored during treatment. In case of elevated blood pressure or headache or dizziness, patients should seek medical advice in a timely manner to receive antihypertensive medication or re-adjustment of the original antihypertensive treatment plan. In such scenarios, the dose of Anlotinib should be adjusted or suspended if necessary. After antihypertensive treatment, patients with persistent high blood pressure or hypertensive crisis or severe hypertensive retinopathy should immediately stop using Anlotinib and receive specialized cardiovascular treatment.

## Conclusions

We report an interesting and rare case of hypertensive retinopathy secondary to oral Anlotinib administration. This report underscores the need to monitor adverse reactions during Anlotinib medication.

## Data Availability Statement

Datasets are available on request: The raw data supporting the conclusions of this article will be made available by the authors, without undue reservation, to any qualified researcher.

## Author Contributions

Conception and design: XZ. Administrative support: QX and LP. Provision of study materials or patients: XZ and QX. Collection and assembly of data: QW and XS. Data analysis and interpretation: QX and LP. Manuscript writing: All authors. Final approval of manuscript: All authors.

## Conflict of Interest

The authors declare that the research was conducted in the absence of any commercial or financial relationships that could be construed as a potential conflict of interest.
